# Association between depressive symptoms and adherence among adolescents living with HIV in the Republic of Congo: A cross sectional study

**DOI:** 10.1097/MD.0000000000021606

**Published:** 2020-08-28

**Authors:** Martin Herbas Ekat, Marcel Yotebieng, Valériane Leroy, Christian Mpody, Merlin Diafouka, Gilbert Loubaki, Dominique Mahambou – Nsondé, Bienvenu Rolland Ossibi Ibara, Charlotte Bernard, Caroline Sabin, Renaud Becquet

**Affiliations:** a Unit of Infectious Diseases, Brazzaville University Hospital, Brazzaville, Congo; b Division of General Internal Medicine, Department of Medicine, Albert Einstein College of Medicine, Bronx, NY, USA; c Division of Epidemiology, College of Public Health, The Ohio State University, Columbus, OH, USA; d Inserm U1027, Université Toulouse 3, Toulouse, France; e Ambulatory Treatment Center of Brazzaville, Brazzaville, Congo; f Inserm, ISPED, Bordeaux Population Health Research Center, UMR 1219, Bordeaux, France; g Institute for Global Health, UCL, London, UK.

**Keywords:** adherence, adolescents, Congo, depression, HIV

## Abstract

The increasing availability of antiretroviral therapy (ART) worldwide is yet to result in decreasing HIV-related mortality among adolescents (10–19 years old) living with HIV (ALHIV) in part because of poor adherence. the poor adherence might itself be due to high level of depression. We assess the prevalence of depressive symptomatology and it's associated with adherence among ALHIV receiving ART care in Brazzaville and Pointe Noire, Republic of Congo (RoC).

Adolescents aged 10 to 19 years, on antiretroviral therapy (ART), followed in the two Ambulatory Treatment Centers (ATC) in Brazzaville and Pointe Noire, RoC were included in this cross-sectional study. From April 19 to July 9, 2018, participants were administered face to face interviews using a standardized questionnaire that included the nine-item Patient Health Questionnaire (PHQ-9). Participants who reported failing to take their ART more than twice in the 7 days preceding the interview were classified as non-adherent. Bivariate and multivariable log-binomial models were used to estimate the prevalence ratio (PR) and 95% confidence interval (95%CI) assessing the strength of association between predictors and presence of depressive symptoms (PHQ-9 score ≥9).

Overall, 135 adolescents represented 50% of ALHIV in active care at the 2 clinics were interviewed. Of those, 67 (50%) were male, 81 (60%) were 15 to 19 years old, 124 (95%) had been perinatally infected, and 71 (53%) knew their HIV status. Depressive symptoms were present in 52 (39%) participants and 78 (58%) were adherent. In univariate analyses, the prevalence of depressive symptoms was relative higher among participants who were not adherent compared to those who were (73% vs 33%; PR: 2.20 [95%CI: 1.42–3.41]). In multivariate analysis, after adjustment for report of been sexually active, alcohol drinking, age category (10–14 and 15–19), not in school, loss of both parents, the association between depression and adherence was strengthened (PR: 2.06 [95%CI: 1.23–3.45]).

The prevalence of depressive symptoms in adolescents living with HIV is high and was strongly associated with poor adherence even after adjustment of potential confounders. Efforts to scale-up access to screening and management of depression among ALHIV in sub-Saharan is needed for them to realize the full of ART.

## 1 Introduction

By the end of 2017, around 1.8 million adolescents (aged 10–19 years) were living with HIV worldwide,^[1]^ the vast majority of whom were residing in sub-Saharan Africa.^[2]^ With the scaling up of access to HIV care and antiretroviral treatment (ART) in this region over the past decade, many children infected perinatally are now surviving to adolescence.^[3]^ However, among adolescents living with HIV (ALHIV), HIV-related mortality has remained constant over time,^[1]^ mainly because of poor adherence to ART. The likely reason for this poor adherence is depression.^[4]^ Though research on mental health among adolescents lags considerably behind that in adults, particularly in resource-limited settings, evidence from high-income settings suggests that adolescents with HIV are at increased risk of mental health challenges including depression.^[5]^ Kim et al reported that almost 1 in 5 adolescents living with HIV in Malawi present with symptoms consistent with depression.^[6]^ Depression is a significant predictor of non-adherence to ART in people with HIV.^[7]^ A systematic review, involving more than 10,000 adults infected with HIV in sub-Saharan Africa, reported that individuals with depression were 55% less likely to have good adherence to ART than those without depression.^[8]^ If the full benefit of efforts to scale-up ART are to be realized among ALHIV in sub-Sahara Africa, effort is needed to better quantify the burden of depression and its impact on adherence in this critical population.

As in adults, an increase in unprotected sexual behavior has been reported in adolescents with depressive symptoms in the United States.^[9]^ Depression among ALHIV in sub-Saharan Africa is associated with being an AIDS orphan,^[10]^ having spent less time in school,^[11]^ having been forced to have sex, being confronted with violence outside the school environment, not feeling safe at home, and not being integrated into a teen community.^[12]^

In Brazzaville, the Republic of Congo (RoC), 3.1% of the population were believed to be living with HIV in 2016.^[3]^ Twenty-three percent of people living with HIV are on ART, almost less than half of whom are followed at one of two Ambulatory Treatment Centers (ATCs) in Brazzaville and Pointe-Noire. Adolescents with HIV in this setting face many barriers, including a lack of knowledge of their own HIV status, their developing sexuality and conflict with host families where the adolescents are, themselves, orphaned.^[13]^ To address these issues, psychological support is offered in both centers with activities such as psychological interviews for the child, either alone or with his/her parents, discussion groups, home visits, family mediations and other, more specific, activities.

Whilst psychological support is the first component of the management of depression in adolescents, no data are available on depression among adolescents living with HIV in RoC.

In this study, we aimed to assess the burden of depressive symptoms among ALHIV and its association with adherence to ART.

## 2 Methods

### 2.1 Design and settings

This cross-sectional study was conducted at the two ATCs in Brazzaville and Pointe-Noire, RoC. The Brazzaville ATC was implemented in 1996, and currently treats 3000 patients; the Pointe-Noire ATC was opened in 2001 and currently treats approximately 4500 patients. Both sites are jointly managed by the Ministry of Health and the Population and the French Red Cross; together they provide care for nearly half of patients living with HIV in the country. As of March 31, 2018, 271 ALHIV were receiving care at one of the 2 centers, 147 in Brazzaville and 124 in Pointe-Noire. All of these ALHIV were on ART according to national guidelines.

### 2.2 Study population

Between April 19 to July 9, 2018, adolescents are known to be actively receiving care at the two participating ATCs were invited either by telephone or during a routine clinic visit to participate in the study. For those who were interested in participating in the study, consent or assent (for children aged 10–15 years by their parents, as recommended by the Ethics Committee) was sought.

### 2.3 Data collection

Face-to-face interviews were conducted to gather information about the adolescent's family situation, knowledge of HIV, exposure to violence and substance use, and adherence to ART. To avoid accidental disclosure of serostatus, any reference to the words “HIV” or “AIDS” was omitted from the questionnaire, and interviewers were trained to avoid any mention of something suggestive except for participants who were already aware of their HIV status.

### 2.4 Measures

Depressive symptoms were elicited using the Patient Health Questionnaire-9 (PHQ-9). The choice of PHQ-9 was motivated by the fact that Lingala translation was readily available from a study among pregnant women in the neighboring Kinshasa region.^[14]^ PHQ-9 includes the nine items from the Diagnostic and Statistical Manual of Mental Disorders 4th edition (DSM-IV) used in the diagnosis of depression. Each item is assigned a score ranging from 0 to 3 depending on the presence and duration of each symptom in the past two weeks: 0 if the symptom was never present, 1 if it had been present several days of the week, 2 if it had been present more than half the time, and 3 if it had been present almost every day. Based on the sum of these scores, depressive symptoms are classified as insignificant (score of 0–4), mild,^[5–9]^ moderate,^[10–14]^ moderately severe^[15–19]^ or severe.^[20–27]^ In the present study, participants with PHQ-9 score ≥9 were classified as having symptoms suggestive of depression. This threshold has been used in another study that has reported on the performance of the PHQ-9 in a similarly aged population.^[15]^ In this study of adolescents aged 13 to 16 years who were hospitalized in a pediatric ward (in Germany), a PHQ-9 score ≥9 was found to have a sensitivity of 82.5% and a specificity of 90.4% for the diagnosis of depression.^[15]^

Adherence to ART was assessed by asking participants or their caregivers if they “have not taken their medication at least once in the last 7 days?”^[16]^ Those who responded affirmatively were then asked ”Was it repeated for several days?“ If the answer was also affirmative, they were asked to report the number of times that this had happened. Adherence was then further categorized as ‘good’ if the medication had not been missed, ‘less good’ if the medication had been missed for one day, ‘bad’ if it had been missed for two days and ‘very bad’ if it had been missed for more than two days.

Other variables considered in this study included: age (dichotomized as 10–14 and 15–19 years), gender (female vs male), living status of parent (both father and mother alive, both deceased, or one parent deceased and the other alive), the availability of family help for attending clinic visits and taking treatment (available for both activities, for one only, or for neither), whether the adolescent had disengaged from care (adolescent had not attended the center within 90 days after a scheduled appointment) at least once since the initiation of ART, the way in which the adolescent had become aware of his/her HIV status (following voluntary testing, through screening following a serious disease, through serological screening after the diagnosis of one or both parents, or the adolescent remains unaware of his/her status) and adherence to ART.

### 2.5 Statistical analysis

Qualitative variables are presented as percentages and were compared between groups using the Chi-square test or Fisher exact test, as appropriate. Continuous variables are presented using the median and interquartile range and were compared between groups using the Student's *t* test or Mann-Whitney U test, as appropriate. Log-binomial models were used to estimate the prevalence ratio (PR) and 95% confidence interval (CI) assessing the strength of the association between depression, adherence and other covariates. When the models did not converge, we used Poisson regression with robust error variance. Covariables that were found to be associated with depression in the univariate analyses (*P* < .20) were included in a multivariable model to estimate adjusted PRs (aPR). Statistical analyses were performed using STATA 14 (College Station, TX). All tests were performed with the significance level set at 0.05 without adjustment for multiple testing unless otherwise indicated.

### 2.6 Ethics committee

This study has been approved by the Committee of Ethics of Health Sciences Research (CERSSA) of the Ministry of Scientific Research and Technological Innovation, under number 017 / MRSIT / IRSSA / CERSSA.

## 3 Results

### 3.1 Characteristics of patients

**Figure 1:**
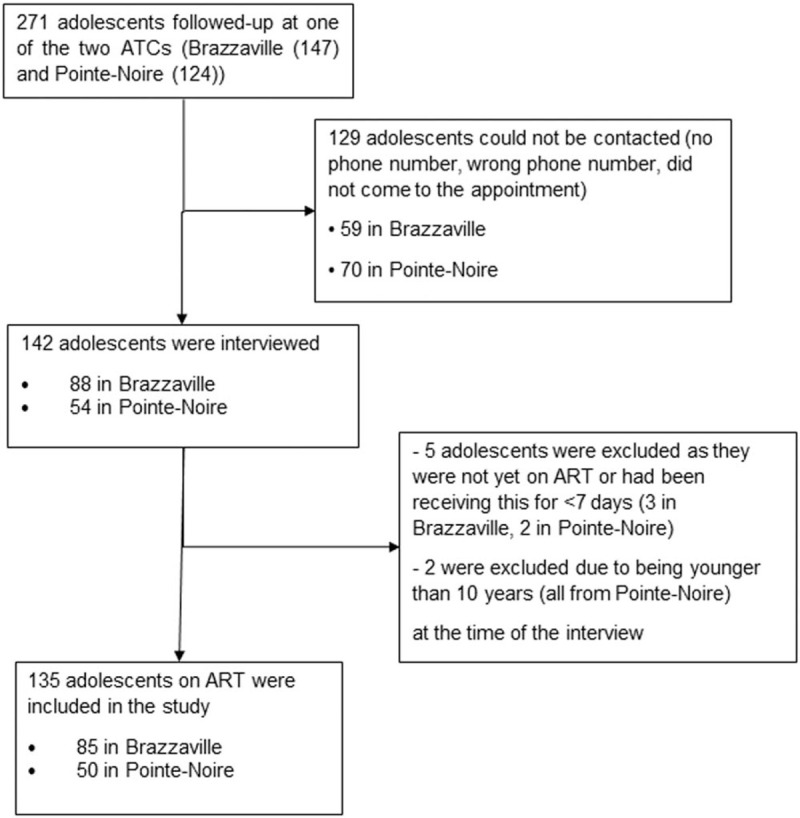
Flow diagram showing the eligibility and recruitment of adolescents to the study.

Of the 271 adolescents under active follow-up at the 2 ATCs, 142 could be contacted and were interviewed. After excluding those who were either not yet on ART or who had not received ART for >7 days, and those were younger than 10 years of age, the final analysis included 135 (50%) of the adolescents, 85 (63%) in Brazzaville and 50 (37%) in Pointe-Noire (Fig. 1). The characteristics of the adolescents included are shown in Table 1. Sixty-seven (50%) were male and 81 (60%) were 15 to 19 years old; 119 (88%) were still in school, of whom 61 (51%) were in high school. Seventy-one (53%) participants were aware of their HIV status. The mode of acquisition of HIV was vertical in 124 (95%) adolescents; discovery of serostatus following a serious/prolonged illness or after voluntary testing occurred in 87 adolescents (64%). Thirty-four (25%) adolescents were sexually active and 11 (8%) reported consuming alcohol. Parents of 49 (36%) adolescents were still alive but 26 (19%) had lost both parents. Fifty-seven adolescents (42%) benefited from support both during their visit to the ATC and while taking ART at home, while 48 (36%) had to undertake both activities alone. Fifteen adolescents (11%) reported having missed ART for ≥3 days in the week before the interview, 14 (10%) had missed scheduled appointment in the three months before the interview, and 12 (9%) had stopped going to the ATC for a period of more than 6 months during follow-up. Sixteen adolescents (12%) had stopped their schooling.

**Table 1:**
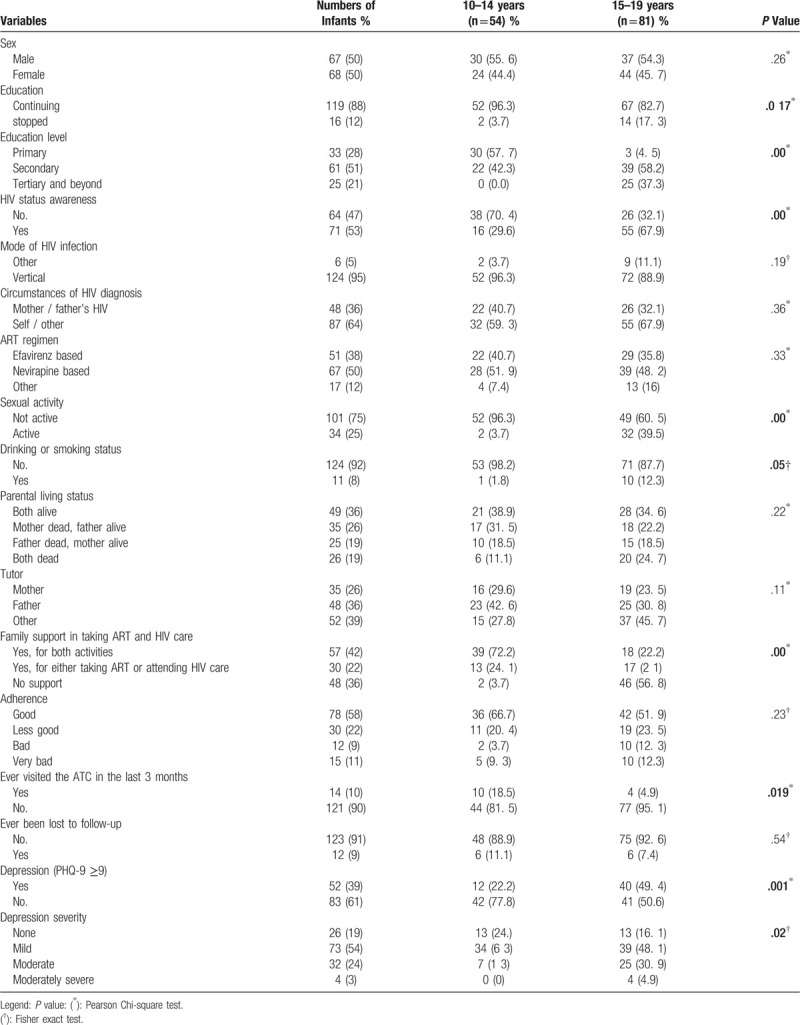
Characteristics of adolescents aged 10 to 19 years at the Ambulatory Treatment Centers of Brazzaville and Pointe-Noire, 2018.

### 3.2 The proportion of depression and adherence

**Figure 2:**
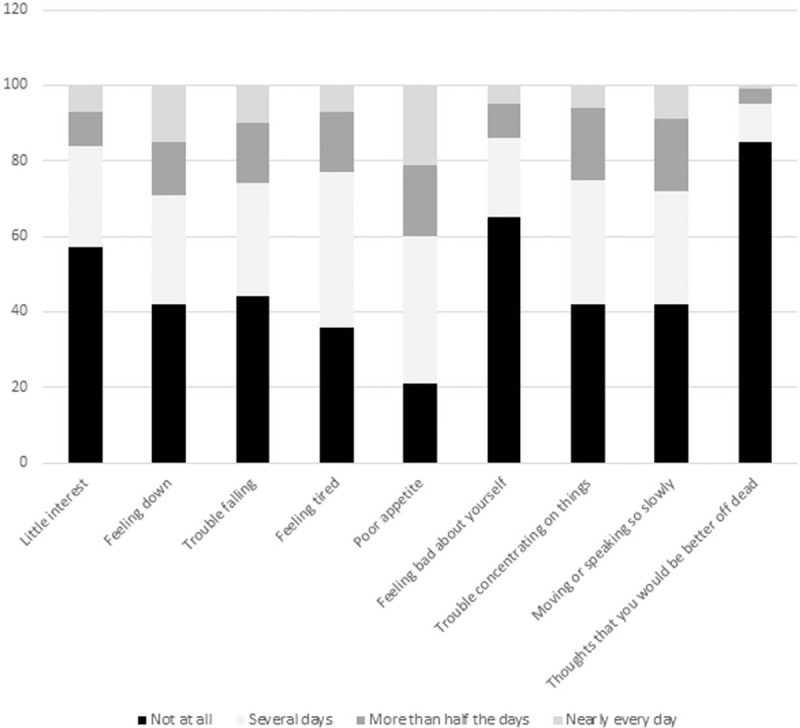
Responses to the 9 questions of the PHQ-9 questionnaire among adolescents aged 10 to 19 years followed at the Ambulatory Treatment Centers of Brazzaville and Pointe Noire in 2018.

Overall, the PHQ-9 score in this group ranged from 0 to 19 (median 7); depressive symptoms were respectively insignificant, mild, moderate and moderately severe in 26 (19%), 73 (54%), 32 (24%) and 4 (3%) ALHIV. Fifty-two adolescents (39%) had a PHQ-9 score ≥9. Figure 2 shows the individual responses to each of the PHQ-9 questions. Adherence was ‘good’, ‘less good’, ‘bad,’ and ‘very bad’ for 78 (58%), 30 (22%), 12 (9%) and 15 (11%) ALHIV respectively.

### 3.3 Factors associated with depression

In univariate, analyses (Table 2), the following factors were associated with depression: age between 15 and 19 years (PR 2.22, 95% CI 1.29, 3.84), having stopped school (PR 1.77, 95% CI 1.13, 2.78), HIV diagnosis following a serious/prolonged illness or after voluntary testing compared after parental diagnosis (PR 1.66, 95% 0.98, 2.79), being sexually active (PR 1.71, 95% CI 1.13, 2.58), drinking alcohol (PR 1.47, 95% CI 0.82, 2.65), having lost both parents compared to having both parents alive (PR 1.44, 95% CI 0.84, 2.49), and not having support for visits to the ATC or for taking ART (PR 1.58, 95% CI 0.98, 2.55). Depression was strongly associated with adherence: the prevalence of depressive symptoms was higher among participants who were not adherent to ART compared to those who were (73% vs 33%; PR 2.20, 95% CI 1.42, 3.41).

**Table 2:**
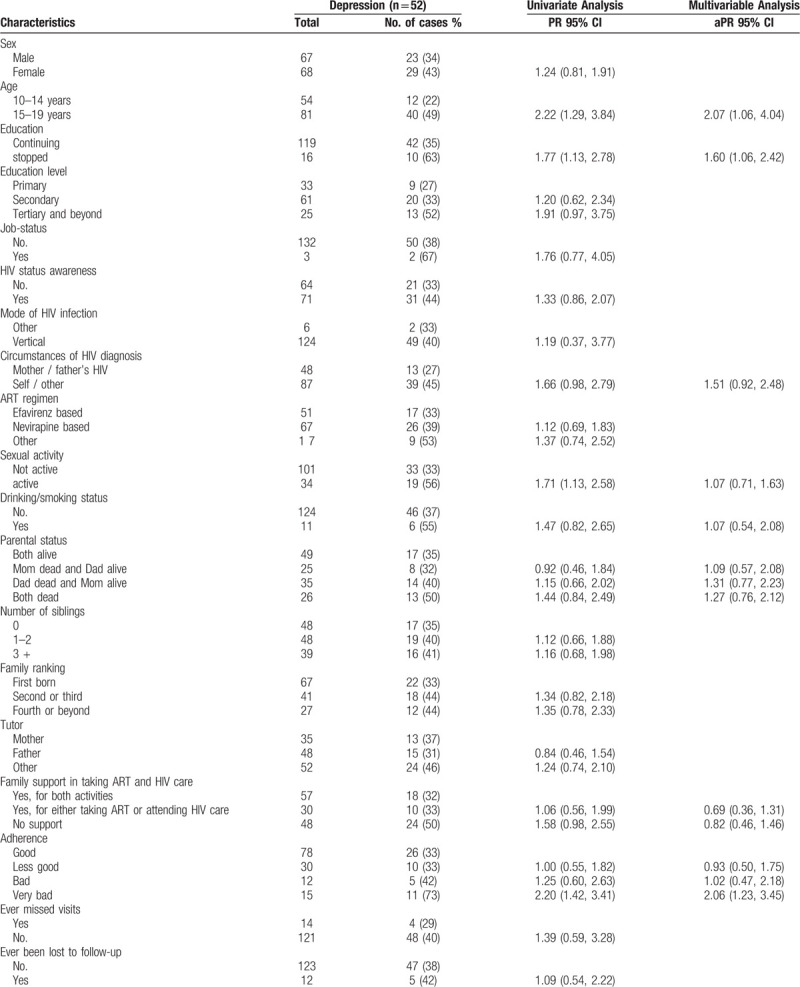
Univariate and multivariable analysis of the association between depression and socio-demographic characteristics of ALHIV enrolled at the Ambulatory Treatment Centers of Brazzaville and Pointe-Noire, 2018.

In multivariate analysis that included those variables found to be associated with depression and adherence in univariate analyses, adolescents who were not adherent were twice as likely to have depressive symptoms compared to those who were (aPR 2.06, 95% CI 1.23, 3.45, Table 2). Adolescents aged 15 to 19 years were more than twice as likely to have depression compared to younger adolescents (aPR 2.07, 95% CI 1.06, 4.04). Those who had stopped school had a prevalence of depression that was 1.60 times (95% CI 1.06, 2.42) as high as that of those who continued to study.

## 4 Discussion

This cross-sectional study is the first to measure depressive symptomatology in Central Africa among adolescents living with HIV on ART. Almost 40% of this population reported symptoms indicative of depression, with higher rates in those aged 15 to 19 years, those who had forgotten to take their ART more than twice in the past week, and those who had stopped schooling.

Few studies have evaluated the prevalence of depression in sub-Saharan Africa in this age group of adolescents with HIV. Studies also reported a high prevalence of depression in ALHIV in Africa.^[6,12,17,18]^ The questionnaire for evaluating depression is different in each of these studies, limiting comparisons, but the high reported rates emphasize the importance of the problem. There are several different tools that are commonly used to evaluate depression. These include screening tools which have a limited number (ranging from 9–21) of questions and can be self-administered (including the PHQ-9, Children's depression inventory (CDI), beck depression inventory (BDI), and the center for epidemiological studies depression (CES-D) scales^[6,19]^) as well as tools that must be used in consultation with a trained psychiatrist or psychologist. The latter is generally used to validate the screening tools and are considered as ”gold standard" reference tools.^[6,20,21]^ Dow et al,^[22]^ in a population of individuals aged 12 to 24 years who were aware of their HIV status and were living with family, reported a prevalence of depression of 12.1% using a threshold of ≥10 on the PHQ-9 questionnaire. Woollett et al,^[12]^ using the Children's Inventory Depression Short Form (CDI-SF), reported a prevalence of 27% in children aged 13 to 19 years in South Africa. Using the SF-CES-D, the prevalence was 25.3% in a population of adolescents aged 15 to 19 years in Zambia^[17]^ and was 26% in adolescents aged 10 to 17 years in Rwanda.^[18]^ Kim et al,^[6]^ using the Children's Rating Scale-Revised (CSD-R), reported a prevalence of 18.9% in children aged 12 to 18 infected with HIV in Malawi. We used the PHQ-9 based on another similar study,^[15]^ but this is the first time it has been used in this population of adolescents with HIV.

We also observed a significantly higher prevalence of depression in our older age group, corroborating previously reported results of a peak in incidence of depression in those aged 15 to 18 years^[23]^ and of a continued increase in the prevalence of depressive symptoms with age.^[24]^ Middle and late adolescence were found to be strongly associated with depressive symptoms corresponding to what has been reported previously.^[23]^ Kim et al^[11]^ reported that older age within the adolescent age group was associated with depression. In Dow et al's study,^[22]^ the risk of depression increased with age. Several reasons may explain the increase of depression in this period of human development including psychological, social and biological development. Indeed, at this time of brain development and changes in cognitive function, adolescents improve their understanding of their surrounding environment and become subject to emotion and stress, and are able to express the effects of these.^[4]^

We found some significant differences in Table 1, there were more adolescents aged 15 to 19 years aware of their HIV status compared to 10 to 14 years old, it could be related that some parents in this cohort postpone the age of the announcement of serostatus to their children. Young children have more support for visits to ATC or for taking ART, compared to adolescents aged 15 to 19, and there were more adolescents aged 10 to 14 years who were not sexually active compared to adolescents aged 15 to 19. These data are very much in line with expectations.

Studies of the mental health of adolescents with HIV must take several factors into consideration, including the changes that occur at puberty and during the transition of the child into an adult, medical issues including knowledge of the HIV status of the individual and his/her access to care, the integration of the adolescent into the community and the influence that the community may have on the behavior of the adolescent, the existence of traumatic events in the history of the adolescent, as well as local environmental factors, including access to and pressure to use abusive substances.^[25]^ In our questionnaire, we incorporated questions relating to schooling, which to some extent represents community, family life, and access to care and ART. We also considered interaction with the care structure by exploring attendance at scheduled appointments, and the use of abusive substances. Stopping school during adolescence was also found to be a factor associated with depressive symptoms in our study. The cessation of schooling in this setting is often a result of lack of support, most often after the loss of parents. The factors related to schooling, were previously mentioned, ranging from the short time remaining in school,^[11]^ evaluated on both a screening tool and a reference tool. Poor adherence to ART was associated with depressive symptoms in this study. For some authors, the fact that doses of ART have been missed may be a sign of depression itself.^[26]^ But the association between depression and ART adherence has been found only in studies where the prevalence of depression was high.^[18,22]^ However, in studies where the level of depression is less than 10%, no association was found, either in children^[27]^ or in adults.^[28]^

Several limitations exist with our study, the first being that it is a cross-sectional study that relies on only a single assessment of depression. A longitudinal study would allow us to confirm the diagnosis of depression over a longer period and would also allow us to determine the direction of any associations seen. For logistical reasons, we were unable to measure the viral load, a good indicator of ART adherence, in order to confirm the relationship with depression.

## 5 Conclusion

This study showed a high prevalence of depression in adolescents with HIV in Central Africa, with the prevalence appearing to increase with age. Factors identified as being associated with a higher rate of depression include those related to the individual, his/her experience of illness and his/her interaction with other members of the community. Interventions to improve the quality of life of adolescents with HIV are needed to ensure optimal outcomes for this particularly vulnerable population in the future.

## Acknowledgments

The authors thank participants of the study and the ATCs staff in Brazzaville and Pointe – Noire, Republic of Congo. The study was conducted as part of the Francoise Barre-Sinoussi Scholarship for MHE.

## Author contributions

The literature review was conducted by MHE, MY and CB. Study design and methods were developed by MHE, MY, VL, RB. Data analysis was done by CM, MY, and MHE. Data interpretation was done by MHE, VL, MY, CB, CS, GL, MD, DMN and BROI.

Writing was completed by MHE, VL, MY, CM, CB, CS, RB. All authors reviewed the final manuscript for submission.
